# Prediction of the histology of colorectal neoplasm in white light colonoscopic images using deep learning algorithms

**DOI:** 10.1038/s41598-021-84299-2

**Published:** 2021-03-05

**Authors:** Seong Ji Choi, Eun Sun Kim, Kihwan Choi

**Affiliations:** 1 Division of Gastroenterology and Hepatology, Department of Internal Medicine, Korea University College of Medicine, 73 Goryeodae-ro, Seongbuk-gu, Seoul, 02841 Republic of Korea; 2 Center for Bionics, Korea Institute of Science and Technology (KIST), 5, Hwarang-ro 14-gil, Seongbuk-gu, Seoul, 02792 Republic of Korea

**Keywords:** Gastroenterology, Colonoscopy

## Abstract

The treatment plan of colorectal neoplasm differs based on histology. Although new endoscopic imaging systems have been developed, there are clear diagnostic thresholds and requirements in using them. To overcome these limitations, we trained convolutional neural networks (CNNs) with endoscopic images and developed a computer-aided diagnostic (CAD) system which predicts the pathologic histology of colorectal adenoma. We retrospectively collected colonoscopic images from two tertiary hospitals and labeled 3400 images into one of 4 classes according to the final histology: normal, low-grade dysplasia, high-grade dysplasia, and adenocarcinoma. We implemented a CAD system based on ensemble learning with three CNN models which transfer the knowledge learned from common digital photography images to the colonoscopic image domain. The deep learning models were trained to classify the colorectal adenoma into these 4 classes. We compared the outcomes of the CNN models to those of two endoscopist groups having different years of experience, and visualized the model predictions using Class Activation Mapping. In our multi-center study, our CNN-CAD system identified the histology of colorectal adenoma with as sensitivity 77.25%, specificity of 92.42%, positive predictive value of 77.16%, negative predictive value of 92.58% averaged over the 4 classes, and mean diagnostic time of 0.12 s per image. Our experiments demonstrate that the CNN-CAD showed a similar performance to that of endoscopic experts and outperformed that of trainees. The model visualization results also showed reasonable regions of interest to explain the classification decisions of CAD systems. We suggest that CNN-CAD system can predict the histology of colorectal adenoma.

## Introduction

Colon cancer is a major cause of morbidity and mortality worldwide^1^. Colon cancer develops in steps from benign polyps through multiple processes over time, and these steps enable the doctors to screen and prevent the cancer before its actual development^2^. During a colonoscopy, which is the most important diagnostic modality for colon cancer screening, endoscopists encounter many abnormal lesions, including premalignant and malignant lesions, and they make a diagnosis with the help of histologic reports from removed tissue^3^. Microscopic analysis has been the basis for cancer diagnosis; however, the formulation of the histologic report takes a few days, and several additional days are required if any special stains are needed. Moreover, many lesions may exist in the colon, although benign lesions are more prevalent, and a histological examination of all lesions is expensive^4^.

To diagnose cancer relatively early, easily, accurately, and economically, many authors have proposed performing an optical biopsy, which predicts the histology of the lesion by its surface features before histological confirmation. In addition, recently developed imaging techniques, such as narrow-band imaging, endocytoscopy, and laser-induced fluorescence spectroscopy have shown promising results^5–7^. However, these newly invented techniques require new endoscopic devices, which further increase the economic burden. Furthermore, the performance of optical biopsies is operator-dependent, and thus, many non-expert endoscopists must depend solely upon the histologic report.

To overcome the aforementioned disadvantages, the objective of our study was to explore the application of deep learning to analyzing white light colonoscopic adenoma images and build a computer-aided diagnostic (CAD) system. Deep learning, the application of which has been expanding in various academic fields, is an optimized tool for automatically extracting features and classifying images, and we considered it an efficient means of achieving our goal. The aim of this study was to develop a CAD system based on deep learning models to support the clinically efficient optical biopsy by predicting the histopathology of colorectal tumors.

## Methods

### Study design and methods

We prepared a dataset by collecting colonoscopic images from Korea University Anam Hospital (KUMC), Seoul, Republic of Korea, and we also prepared a separate dataset by collecting images from Hanyang University Hospital (HYUMC), Seoul, Republic of Korea. The colonoscopic images were taken using a standard endoscope (CF-H260AL, CF-Q260AL, CF-H290L, or CF-HQ290L; Olympus Medical Systems, Co. Ltd., Tokyo, Japan) and a standard endoscopic system (EVIS LUCERA ELITE CV-260/CLV-260 or CV-290/CLV-290SL; Olympus Medical Systems, Co. Ltd., Tokyo, Japan). All the colonoscopic images were automatically stored in the hospital Picture Archiving and Communication System (PACS).

**Figure 1:**
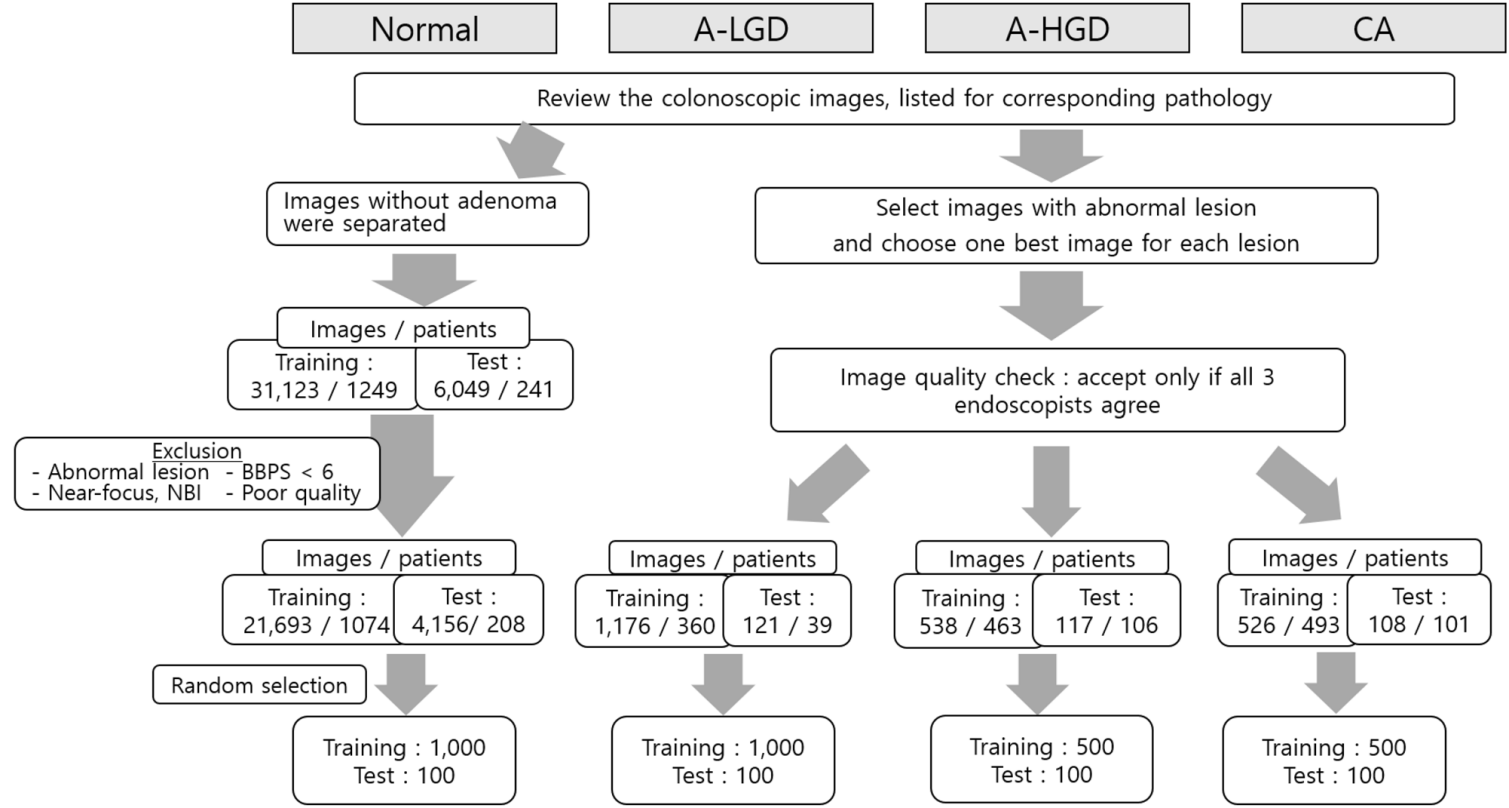
Flowchart of the data collection. Images were collected until they met the target number of images for each class in the training and test sets. *CA* adenocarcinoma, *A-HGD* adenoma with high grade dysplasia, *A-LGD* adenoma with low grade dysplasia.

Figure 1 shows the flow diagram of how the image collection was performed. The images were labeled into 4 different categories including normal, adenoma with low grade dysplasia (A-LGD), adenoma with high grade dysplasia (A-HGD), and adenocarcinoma (CA) based on the histologic report. The adenoma in the images included tubular adenoma, tubulovillous adenoma, villous adenoma and serrated adenoma. The KUMC dataset consists of 1000 for normal and A-LGD, and 500 for A-HGD and CA, and the HYUMC dataset consists of 100 for balancing the number of images for each class.

Because not all colonoscopic procedures contain images of adenoma, a list was made for colonoscopies with adenoma from January 2017 to February 2020. Only one procedure per patient is included, and the collection of images for each category was stopped when the target number was reached.

For A-LGD, H-LGD, and CA, we included standard white light colonoscopic tumor images that had been histologically confirmed. Especially for A-LGD and A-HGD, images were included only if they had been removed with en-bloc resection, because biopsy results cannot always be trusted if only some parts of the tumor were analyzed. As in previous studies, poor quality images, which were defined as images with motion-blurring, out-of-focus, insufficient brightness, or a considerable amount of blood, mucus, or fecal material covering the tumor’s surface, were excluded^8,9^. Images captured using an advanced technique, such as near-focus mode or narrow-band imaging (NBI), were also excluded. Colonoscopic images that were collected and had no adenoma were gathered to be included in the normal category. For the normal category, images without adenoma among the reviewed images were included, and colonoscopic images with abnormal lesion, Boston Bowel Preparation Score lower than 6, near-focus mode, NBI, and previously defined poor-quality images were excluded. Abnormal lesion included uneven surface caused by hyperplastic polyp, inflammatory polyp, hamartomatous polyp, submucosal tumor, and external compression.

Three experienced endoscopists with more than 5 years of colonoscopy experience participated in collecting data and reviewed the images starting from January 2017 sequentially. Only one best image was chosen per lesion, and the image was not selected if any of the endoscopists considered the image to be of poor quality. The data collection was finished when each class reached the target number of images.

The study protocol was approved by Korea University Anam Hospital Institutional Review Board (2019AN0424), and the need for informed consent was waived by the Institutional Review Board due to the retrospective nature of the study. The authors confirm that all the experiments were performed in accordance with relevant guidelines and regulations. All patient data were anonymized, and all images were de-identified and organized into a different order.

The proposed CAD system used ensemble learning with three CNN models, which transferred the knowledge of digital photography and learned with colonoscopic images to classify the images into one of 4 different pathologic categories. The CAD system also revealed heatmaps of the relevant regions with respect to the predicted class. The details are explained in the following subsections.

### Colonoscopic image datasets preparation

**Figure 2:**
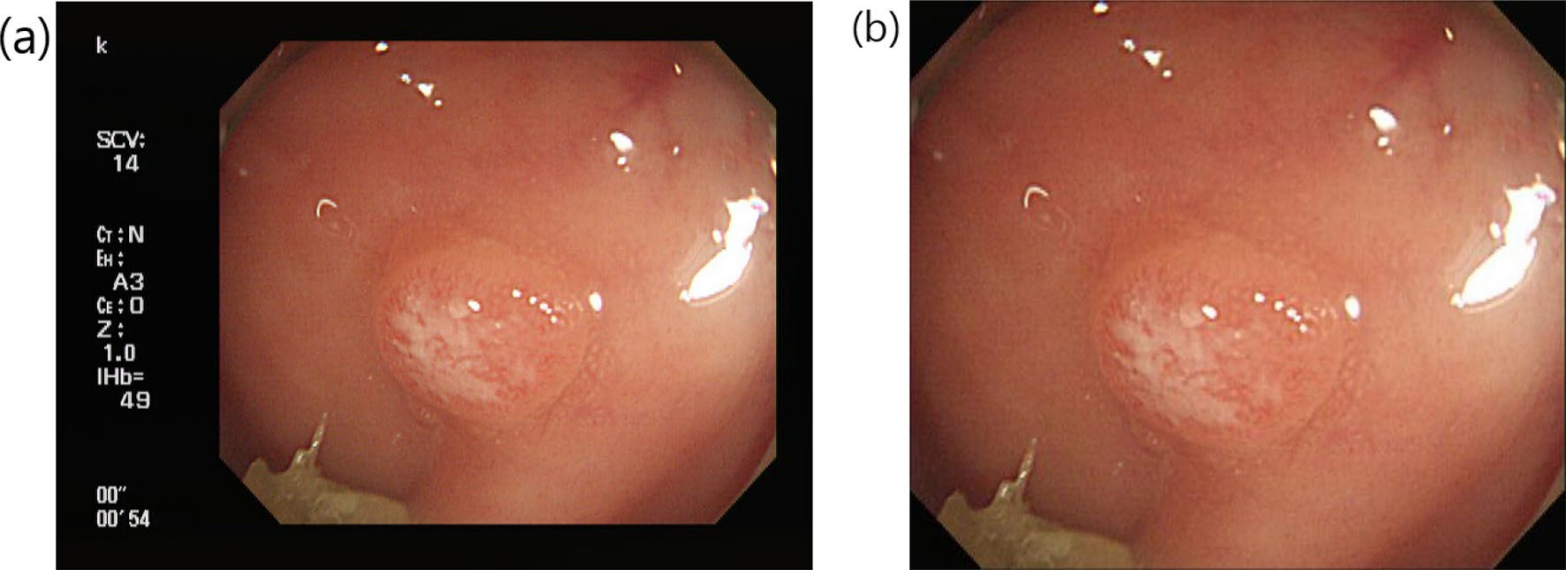
Original colonoscopic image (**a**) stored in the hospital Picture Archiving and Communication System (PACS) and the cropped and resized image (**b**) for training and testing our convolutional neural network-computer-aided diagnostic system.

The collected colonoscopic images originally had uneven black areas around the endoscopy camera captures to display system information. In addition, the original colonoscopic images were in various resolutions due to the different acquisition systems and settings. In order to standardize our dataset, we cropped the colonoscopic images to exclude unnecessary black areas and resized the cropped images into 480 × 480 (Fig. 2).

### Network architectures

Recently, deep learning has become a dominant machine learning tool in the field of visual recognition. Convolution neural networks (CNNs) showed human-level image classification accuracy in the ImageNet Large Scale Visual Recognition Challenge^10–14^. In this study, we implemented the CNN-based CAD system (CNN-CAD) based on contemporary CNN architectures and comparatively evaluated their diagnosis performance. For CNNs, we used Inception-v3^12^, ResNet-50^13^, and DenseNet-161^14^ as baseline models (Supplementary Table 1). To adapt the existing CNN models for colorectal diagnosis, we replaced the last fully connected layer, which predicts 1000 ImageNet categories, with a fully connected layer for predicting 4 categories (normal, A-LGD, A-HGD, and CA). In addition, we removed the auxiliary fully connected layer of the Inception-v3 model and replaced it with a fully connected layer for predicting the 4 categories.

### Balanced sampling for imbalanced data

The frequencies of the 4 different categories are uneven in our colonoscopic dataset. For example, if we randomly select an image from the dataset, the probabilities that the image is labelled with normal or adenocarcinoma differ. To avoid overfitting the models with the samples from frequent categories, we rebalanced sampling weights inversely proportional to the frequencies of the categories. When input images were sampled with the rebalanced sampling weights, the probabilities that input images belong to the categories were the same.

### Training deep neural networks

All the neural networks were initialized with models pre-trained with the ImageNet dataset^15^. Because the ImageNet dataset contains physical objects, the model could not be directly trained for colonoscopic images. As discussed above, the final dense layers of the models were replaced by new layers specific to the task of diagnosing colorectal tumor.

The deep learning models were retrained end-to-end to fine-tune the weights of the networks. To augment the data, we randomly flipped the input images horizontally and vertically. For training, we used a stochastic gradient descent (SGD) optimizer with a learning rate of 10^−4^, momentum of 0.9, and batch size of 8. The neural networks were fine-tuned using during 300 epochs. In order to update the model parameters, the colonoscopic images were sampled from the training set according to the balanced sampling rule. During each epoch, the same number of images in the training set were sampled and fed into the neural networks. The codes were written in PyTorch running on an NVIDIA Titan Xp GPU^16^.

### Ensemble learning for computer-aided diagnosis

**Figure 3:**
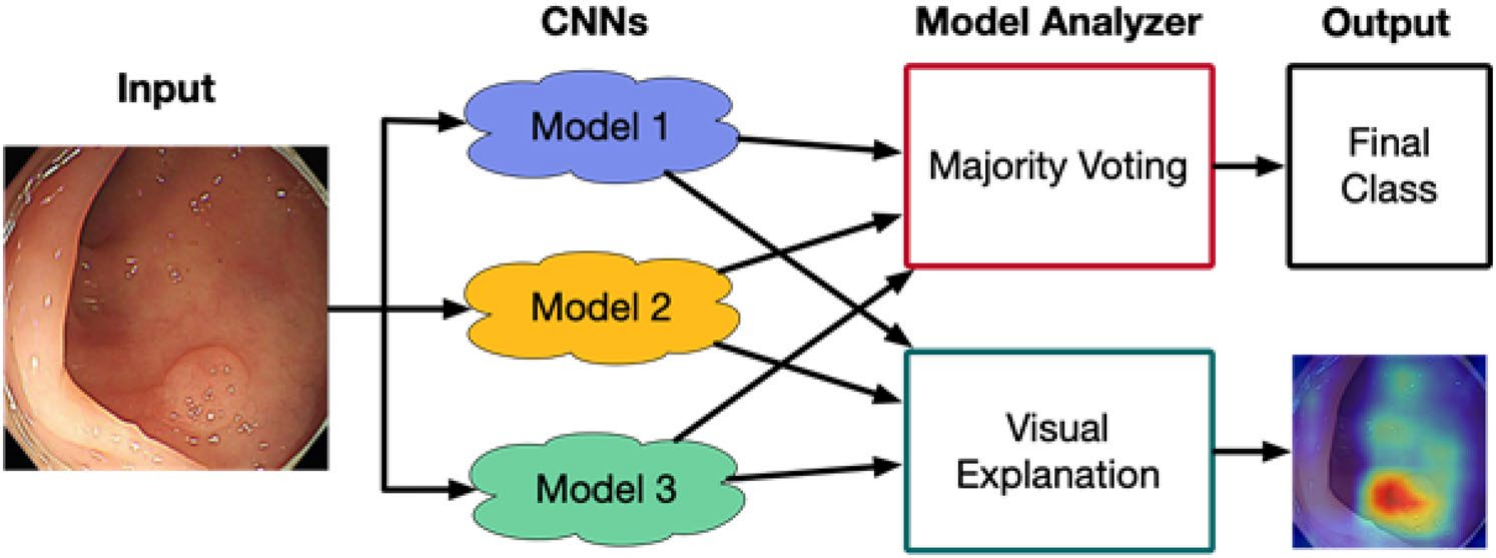
Overview of computer aided diagnosis (CAD) system based on 3 CNN models. Each CNN model independently predicts the class of input colonoscopic image. The following model analyzer decides final class from the predictions and provides the corresponding visual explanation.

As shown in Fig. 3, our CNN-CAD system collected the decisions from multiple CNN models for its output. In order to combine the predictions from the CNN models into one final prediction, we utilized ensemble learning by combining the models in parallel. Although we were able to combine hard-decisions from the models, we combined soft-decisions by averaging the last activation maps among the models. Making a final decision based on soft-decisions rather than hard-decisions obtained better predictive accuracy. By selecting the most probable class, we were able to retrieve the mode of multiple CNN models.

### Visualization of CNN-CAD decision

A recently proposed technique called Class Activation Mapping (CAM) enables CNNs to highlight the regions used by a restricted class of image classification^17^. In this study, we employed Grad-CAM, which allowed us to visualize the predicted class scores of any given image without the need for architectural changes, retraining processes, or any additional network components^18^. The heatmap reveals the relevant image regions and the CNN-CAD makes a decision. We further compared the resultant heatmaps among the different CNN-CAD models.

### Performance evaluation using tenfold cross-validation

In this subsection, we assess the histopathological prediction performance of our deep learning model with the KUMC dataset. In general, the performance of a machine learning model is warranted if the model has a low prediction error, which can be directly calculated by applying the model to predict the response to new data that were not used in the training process. Although the prediction error can be easily calculated if a designated test set is available, our colonoscopic data of colorectal tumors and histopathology were limited. In the absence of a large test set, cross-validation can be used for model assessment as well as model selection^19^. By holding out a subset of the data set to prepare a validation set, and then applying the deep learning model to the validation set, which was not used for training, cross-validation can estimate the prediction error in order to evaluate the performance of the model.

In order to evaluate the CNN-CAD system without overfitting, we performed tenfold cross-validation with the KUMC dataset by randomly partitioning the dataset into 10 splits. Each split included 1/10 of the dataset (100 normal images, 100 A-LGD images, 50 A-HGD images, and 50 CA images). In each experiment, we first selected one split as the validation set. We trained the neural networks with the remaining 9/10 of the dataset as the training set. Then, we evaluated the neural networks with the validation set. The experiment was repeated 10 times, where each time a different split was selected as the validation set. Because the 10 splits were mutually exclusive, the models in the 10 experiments predicted the categories for all images in the dataset. Hence, each model was trained and selected independently of the corresponding validation split.

### Multi-center study for testing deep neural networks

Although cross-validation theoretically estimates the expected prediction error, deep learning models are often applied to clinical data from different centers in practice. Furthermore, the properties of colonoscopic images such as brightness and resolution vary depending on clinical environments such as equipment and protocols. In order to further evaluate the performance of our CAD system with more realistic data/situations, we performed a multi-center study by using separate datasets, the KUMC dataset for training and the HYUMC dataset for testing our CNN-CAD system. First, we fine-tuned the three deep learning models with the KUMC dataset during 300 epochs where each epoch in training with 3000 images took approximately 30 s for Inception-v3, 20 s for ResNet-50, and 50 s for DenseNet-161. Then, we tested the trained CNNs and CNN-CAD system with the HYUMC dataset which consists of 400 images including 100 images for each category.

### Quantitative analysis

The primary metric to evaluate the results in the experiments was classification accuracy; we also calculated sensitivity, specificity, positive predictive value (PPV), negative predictive value (NPV), and mean diagnostic time per image (MDT). We used the following definitions in our evaluation. Accuracy was defined as the ratio of the number of correctly classified images to the total number of images. Sensitivity was defined as the ratio of the number of true-positive images to the number of true-positive and false-negative images. Specificity was defined as the ratio of the number of true-negative images to the number of true-negative and false-positive images. The PPV was defined as the ratio of the number of true-positive images to the number of true-positive and false-positive images. Finally, the NPV was defined as the ratio of the number of true-negative images to the number of true-negative and false-negative images.

Since we categorize the colonoscopic images into 4 classes, “true-positive” implies that the predicted class corresponds to the ground truth histology. Hence, we calculated quantitative metrics for each class and averaged the quantitative metrics over the 4 classes. We further compared the outcomes of the three CNN-CADs and of two endoscopist groups having different levels of clinical experience.

### Diagnostic assessment by endoscopist

A test set of 200 images was created by randomly selecting 50 images from each class. Four experts with more than five years’ experience in colonoscopy and six trainees with less than two years’ experience in colonoscopy participated in the classification trial. The participating endoscopists had been trained or were currently in a fellowship program at tertiary university hospitals. The annual colonoscopy volumes for the experts and trainees were more than 1000 and 500, respectively. The classification label of each image was not revealed to the participants. They were asked to classify each image of the test set into one of four classes: normal, A-LGD, A-HGD, or CA.

## Results

### Data collection

Figure 1 shows the number of images collected for the analysis in each class. For A-LGD, a total of 1176 and 121 images were reviewed from 360 and 39 consecutive colonoscopic procedures with A-LGD, and the collection of images was stopped when A-LGD class reached 1000 and 100 images from KUMC and HYUMC, respectively. For A-HGD, a total of 538 and 117 images were reviewed from 463 and 106 consecutive colonoscopic procedures with A-HGD, and the collection of images was stopped when A-HGD class reached 500 and 100 images in KUMC and HYUMC, respectively. For CA, a total of 526 and 108 images were reviewed from 493 and 101 consecutive colonoscopic procedures with CA, and the collection of images was stopped when CA class reached 500 and 100 images from KUMC and HYUMC, respectively. For the normal category, colonoscopic images without adenoma were separated, and 31,123 and 6049 images were reviewed from 1249 and 241 consecutive colonoscopic procedures in KUMC and HYUMC, respectively. After exclusion, 1000 and 100 images were randomly selected among 21,693 and 4156 images from KUMC and HYUMC, respectively.

### Cross-validation results with KUMC dataset

**Figure 4:**
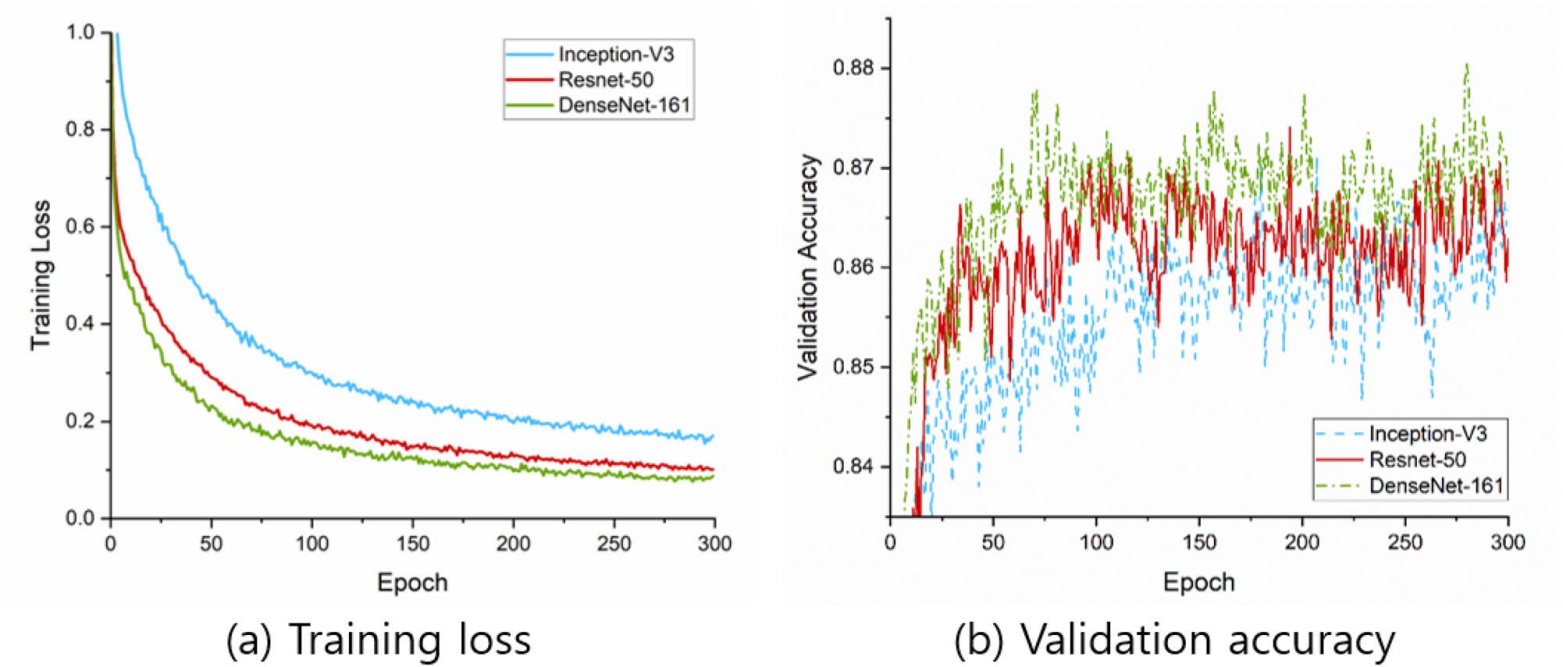
Learning curves of tenfold cross-validation with the KUMC data set. (**a**) Training loss. (**b**) Validation accuracy. The training loss is averaged over the 10 cross-validation models for each CNN and each epoch. The validation accuracy is calculated by evaluating the 10 cross-validation models and collecting the validation results for each CNN and each epoch.

The KUMC dataset consists of 3000 images and 4 categories (1000 normal, 1000 A-LGD, 500 A-HGD, and 500 CA images). Table 1 shows the characteristics of the colonoscopic images. In Fig. 4, the learning curves of tenfold cross-validation are shown. For each epoch, the training loss was averaged over the 10 cross-validation models of each CNN. The validation accuracy was calculated by evaluating the 10 cross-validation models of each CNN for each epoch.

**Table 1:** Characteristics of colonoscopic images.

	Normal (n = 1000)	A-LGD (n = 1000)	A-HGD (n = 500)	CA (n = 500)
**Location**				
Rectum	183	163	97	110
Sigmoid colon	167	188	113	134
Descending colon	166	111	57	49
Transverse colon	192	220	103	87
Ascending colon	224	275	112	107
Cecum	68	43	18	13
**Morphology (A-LGD/A-HGD)**				
Ip		27	11	
Is		354	139	
IIa		611	347	
IIb		8	3	
**Adenoma**				
Tubular		807	363	
Tubulovillous		156	108	
Villous		0	5	
Serrated		37	24	
**Morphology (CA)**				
Protruded				108
Depressive				134
Ulcerative				175
Laterally spreading				83

*CA* adenocarcinoma, *A-HGD* adenoma with high grade dysplasia, *A-LGD* adenoma with low grade dysplasia.

**Figure 5:**
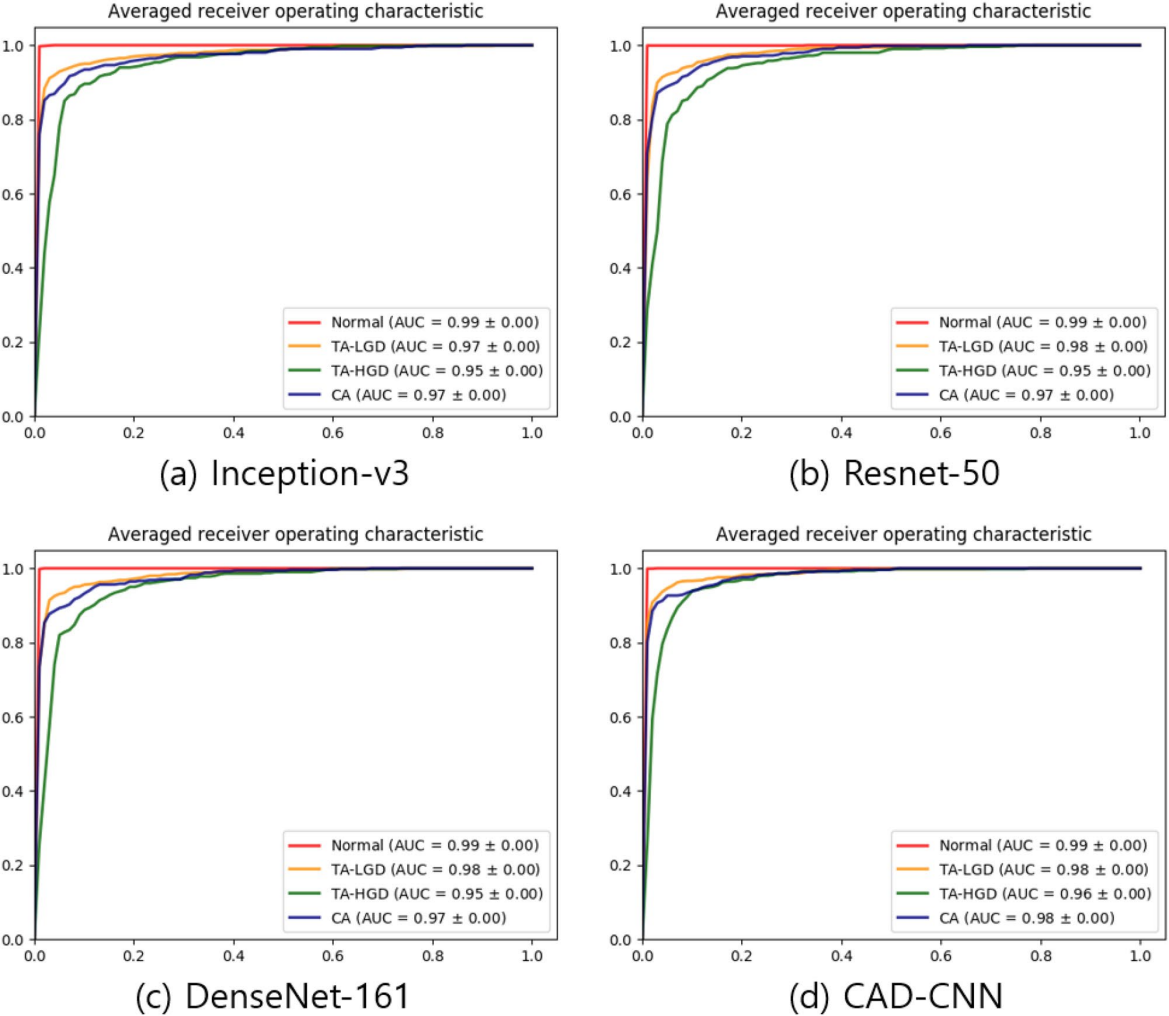
Receiver operating characteristics (ROCs) curves of tenfold cross-validation with the KUMC data set. The 10 cross-validation models which show the best validation accuracy during 300 training epochs are evaluated. The area under curve (AUC) is also listed for each category. (**a**) Inception-v3. (**b**) Resnet-50. (**c**) DenseNet-161. (**d**) CAD-CNN.

For each experiment, we collected the cross-validation result of the best validation accuracy model, which shows the highest validation accuracy during 300 epochs. In Fig. 5, the receiver operating characteristic (ROC) curves of the best validation accuracy models are constructed by plotting the true positive rate (sensitivity) as a function of the false positive rate (1 − specificity). The value of the areas under the curve of ROCs (AUC) ranges from 0.95 to 0.99.

Table 2 shows the performance of the CNN models. The ensemble learning CNN-CAD model showed the best performance in our cross-validation with a F1-score of 0.9055, and its 4-class averaged sensitivity, specificity, PPV, and NPV were 90.65%, 97.57%, 90.55%, and 97.52%, respectively. The cross-validation results of the CNN models are attached as Supplementary Tables 2–5.

**Table 2:** Cross-validation results with performance comparison among the evaluated deep learning algorithms and endoscopists.

	Sensitivity (mean) (%)	Specificity (mean) (%)	PPV (mean) (%)	NPV (mean) (%)	F1-score (mean)	MDT, s
Inception-V3	89.05	97.16	88.88	97.11	0.8894	0.04
ResNet-50	89.98	97.34	89.77	97.27	0.8977	0.03
DenseNet-161	89.88	97.37	89.79	97.35	0.8982	0.05
CNN-CAD	90.65	97.55	90.57	97.52	0.9055	0.12
Endoscopist, expert (mean)	85.00	95.00	85.74	95.05	0.8508	7.96 ± 4.80
Endoscopist, trainee (mean)	77.98	92.63	78.39	92.76	0.7779	9.55 ± 6.26

*NPV* negative predictive value, *MDT* mean diagnostic time per image, *PPV* positive predictive value, *s* seconds.

Table 2 also compares the classification performance of the CNN models and the endoscopists. The endoscopists classified colon adenoma with an overall sensitivity of 80.77%, specificity of 93.58%, PPV of 81.00%, NPV of 93.62%, and MDT of 8.91 s. Among the endoscopists, the expert group showed higher-level results than the trainee group in all parameters: sensitivity (85.00% vs. 77.97%), specificity (95.00% vs. 92.63%), PPV (85.67% vs. 77.93%), NPV (95.03% vs. 92.70%), F1-score (0.8508 vs. 0.7779), and MDT (7.96 s ± 4.80 s vs. 9.55 s ± 6.26). More importantly, our model showed better results than the expert group in all parameters: sensitivity (90.65% vs. 85.00%), specificity (97.57% vs 95.00%), PPV (90.55% vs. 85.67%), NPV (97.52% vs. 95.03%), F1-score (0.9055 vs. 0.8508), and MPT (0.05 s vs. 7.96 s). Total outcome measures of each endoscopist are listed in Supplementary Table 5.

**Figure 6:**
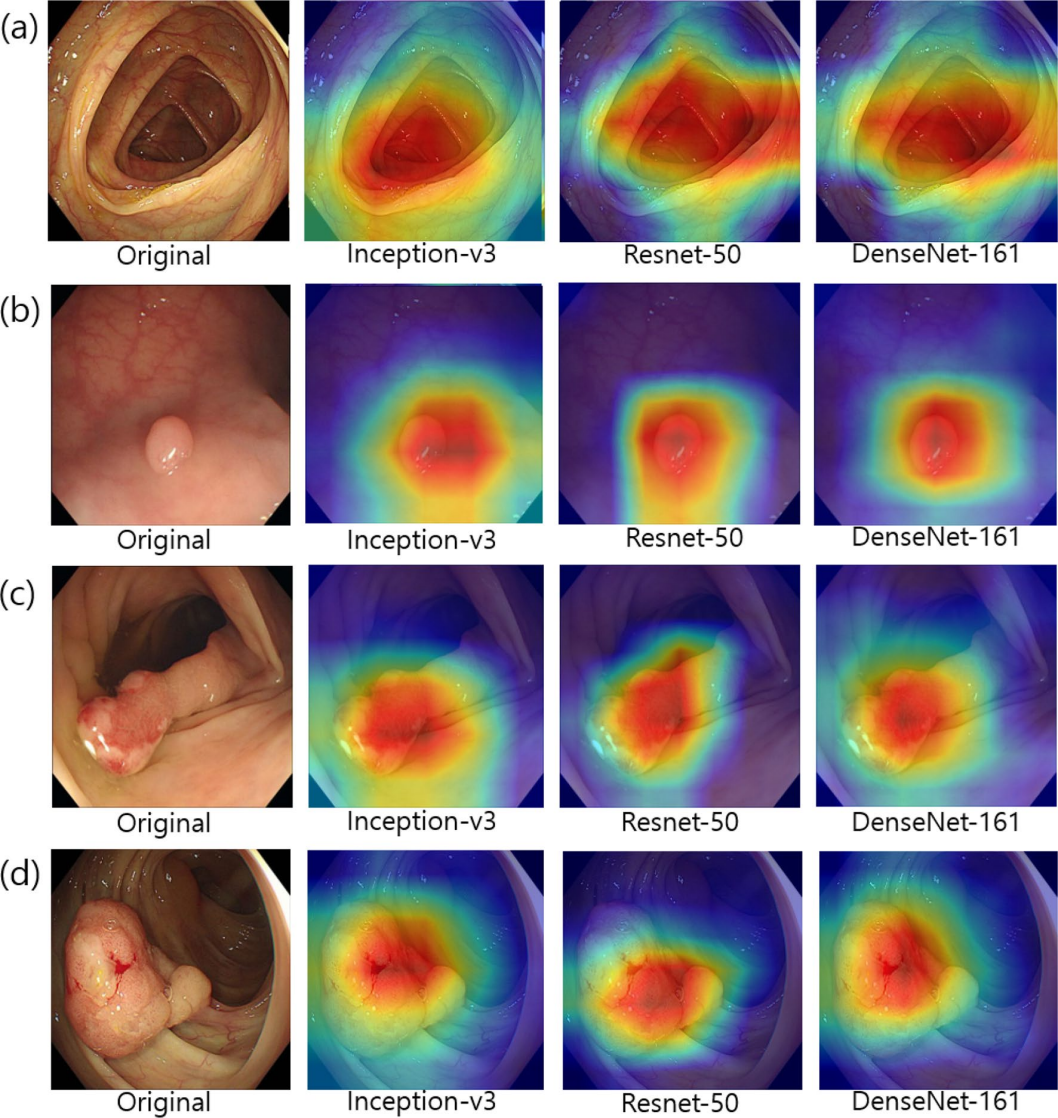
Classification activation maps using different convolutional neural network models in colonoscopic images. (**a**) normal, (**b**) low grade dysplasia, (**c**) high grade dysplasia, (**d**) adenocarcinoma.

Figure 6 shows the CAM in each class using the different CNN models. The CAM highlights the class-specific regions of images and thus helps us visualize the location of the colonoscopic lesions and verify the decision of the CNN-CAD. By means of the CAM, we obtained the visual explainability of the classification yielded by the CNN-CAD.

### Test results with HYUMC dataset

The HYUMC dataset consists of 400 images and 4 categories (100 normal, 100 A-LGD, 100 A-HGD, and 100 CA images). As the same with the cross-validation, we used the best validation accuracy models, and we constructed an ensemble model using the 10 best validation accuracy models for each CNN. Our ensemble learning CNN-CAD system used the 30 best validation accuracy models of the three CNNs to predict the final decision. Table 3 summarizes the test results of CNN models. Our CNN-CAD system based on ensemble learning showed the best performance in our classification test with a F1-score of 0.7681, and the 4-class averaged sensitivity, specificity, PPV, and NPV were 77.25%, 92.42%, 77.16%, and 92.58%, respectively.

**Table 3:** Test result with performance comparison among the evaluated deep learning algorithms and endoscopists.

	Sensitivity (mean) (%)	Specificity (mean) (%)	PPV (mean) (%)	NPV (mean) (%)	F1-score (mean)	MDT, s
Inception-V3	72.75	90.92	72.28	91.07	0.7227	0.04
ResNet-50	74.00	91.33	73.35	91.61	0.7320	0.03
DenseNet-161	74.50	91.50	74.70	91.62	0.7415	0.05
CNN-CAD	77.25	92.42	77.16	92.58	0.7681	0.12
Endoscopist, expert (mean)	72.38	90.58	71.38	90.89	0.7187	7.72 ± 4.11
Endoscopist, trainee (mean)	62.50	86.47	61.91	87.08	0.6217	8.13 ± 4.82

*NPV* negative predictive value, *MDT* mean diagnostic time per image, *PPV* positive predictive value, *s* seconds.

**Figure 7:**
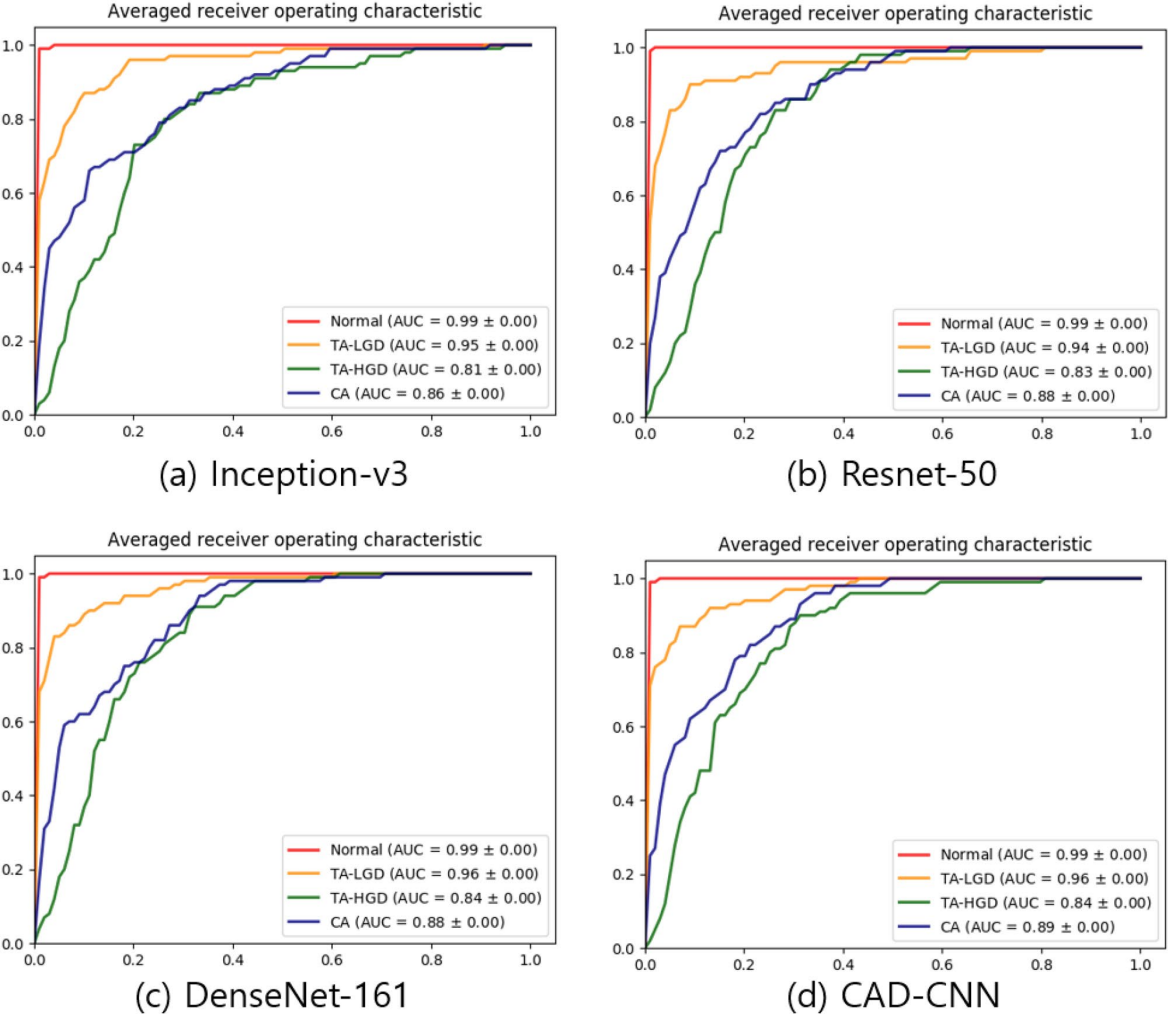
Receiver operating characteristics (ROCs) curves of test results with the HYUMC data set. The 10 CV models which show the best validation accuracy during 300 training epochs with the KUMC data set are evaluated. The area under curve (AUC) is also listed for each category. (**a**) Inception-v3. (**b**) Resnet-50. (**c**) DenseNet-161. (**d**) CAD-CNN.

Table 3 also compares the classification performance of the deep learning models and the endoscopists. The endoscopist experts classified colon adenoma with an overall sensitivity of 67.44%, specificity of 88.53%, accuracy of 67.44%, PPV of 66.65%, NPV of 88.99%, and MDT of 7.92. Among the endoscopists, the expert group showed higher-level results than the trainee group in all parameters: sensitivity (72.38% vs. 62.50%), specificity (90.58% vs. 86.47%), PPV (71.38% vs. 61.91%), NPV (90.89% vs. 87.08%), F1-score (0.7187 vs. 0.6217), and MDT (7.72 s ± 4.11 s vs. 8.13 s ± 4.82). Our CNN-CAD model showed slightly better results than the expert group in all parameters: sensitivity (77.25% vs. 72.38%), specificity (92.42% vs 90.58%), PPV (77.16% vs. 71.38%), NPV (92.58% vs. 90.89%), F1-score (0.7681 vs. 0.7187), and MPT (0.12 s vs. 7.72 s). Total outcome measures of each endoscopist are listed in Supplementary Table 6. The receiver operating characteristic (ROC) curves of the best validation accuracy models are plotted in Fig. 7.

## Discussion

In the field of gastroenterology, colonoscopy is the area where artificial intelligence (AI) is most widely and actively applied. Current researches are focused on detection and classification of colonoscopic lesions^20^, and in this study we applied deep learning to the classification of colonoscopic images into 4 categories. Our model using state-of-the-art CNNs could differentiate the 4 classes successfully. In comparison with that of endoscopists, our model’s performance was similar to that of endoscopic experts and outperformed that of trainees. To the best of our knowledge, this is the first study to demonstrate that a trained AI model can classify white light colonoscopic images into 4 categories (normal, A-LGD, A-HGD, and CA), where most current studies have applied a binary classification (non-neoplastic vs. neoplastic or hyperplastic vs. neoplastic) using enhanced images^8,21–24^.

Overall outcomes of our study decreased in test results with the HYUMC dataset compared to the cross-validation results with the KUMC dataset. From the performance difference between validation and test results, one may suggest that the deep learning models are overfitted. However, we argue that the performance difference is due to the statistical difference between the validation and test sets rather than model overfitting. Since the prediction accuracy of endoscopists also showed a decrease up to 15%, we can observe that the images in the KUMC dataset are easier to predict compared to those in the HYUMC dataset. The learning curves in Fig. 4 with stable training loss and validation accuracy also differs from those of overfitting models. Nevertheless, our model showed similar or slightly better results with experts in the test results, which suggests that our model will perform well in many different situations, and we expect the outcome would improve if we train our model with images from different settings.

The prediction of the histology of colon tumors is quite challenging due to various features of the lesion itself and the nature of the organ where the lesion develops. Even for the same tumor, there are inter- and intra-variances of brightness, size, shape, and texture of the colon lesions. Moreover, the characteristics of the colon, which is a long mobile tube-like organ with angulation and haustra that frequently changes its diameter, make the features of the lesion more variable^25^. To overcome these difficulties and evaluate the lesion more precisely, many imaging techniques, such as NBI, I-Scan, Fujifilm Intelligent Color Enhancement (FICE), confocal laser endomicroscopy, and endocytoscopy, have been developed and have shown good accuracy in many studies^26–29^. To increase the accuracy of AI in the detection or differentiation of colon adenoma, most researchers have applied these virtual chromoendoscopy techniques^22–24,30–33^. However, these techniques are not available in many medical centers, especially in community-based hospitals, and endoscopists who are equipped with these endoscopes require training or education, as long as 6 months, to reach certain levels of competence and accuracy in using them^34,35^. To be able to use these techniques, there are clear diagnostic thresholds and requirements^36^. Our study was designed to overcome these disadvantages and maximize our model’s clinical use by using solely unmagnified white light colonoscopic images. Our CNN-CAD model was able to classify these images successfully.

Compared to many previous studies, we classified images into normal, A-LGD, A-HGD, and CA to aid in planning for the treatment and follow-up in advance. For treatment, endoscopic removal should be primarily considered for A-LGD and A-HGD lesion, and accurate diagnosis could help in deciding proper removal plan, including simple resection, endoscopic mucosal resection or endoscopic submucosal resection. In addition to endoscopic treatment, surgery or palliative treatment could also be considered in CA depending on its stage. We built our model for optical biopsy with artificial intelligence to reduce an unnecessary procedure, save the time delay from discovery of lesion to treatment, and aid endoscopists in making proper treatment decision.

With minor adjustments, we expect our model to achieve higher classification accuracy when enhanced-technique images are used and we will produce a validation image set with these images for the follow-up study. Our model showed high-level sensitivity and accuracy in classifying normal and A-LGD images, but rather low-level sensitivity and accuracy in classifying A-HGD and CA (Supplementary Tables 2–5). In particular, because intramucosal carcinoma (Tis) and CA are differentiated by the depth of invasion, the differentiation of A-HGD and CA was especially challenging for both our model and the endoscopists. Moreover, images of these intermediate stages are relatively rare, and we need to collect more images if we intend to differentiate them further.

Our model is not a definitive tool to make the diagnosis, but it can be used as a helpful tool in predicting the histology of colon lesions and forming treatment plans. The diagnostic accuracy of endoscopic biopsy for colon cancer can be as low as 78.1% owing to many factors, including tumor heterogeneity and the number of biopsies. Our model can help physicians make a more accurate diagnosis by providing a second opinion on re-biopsy or multiple biopsies of colon adenoma, because our data include the pathologic report from en-bloc resections only^37^. Due to these qualities, our model can help endoscopists, especially those who are inexperienced, in quality improvement. Because endoscopists capture the images of the colon tumor when they observe it and CNN models take only 0.03–0.05 s per image for prediction in our study, our CNN-CAD model, which predicts images rather than videos, can diagnose a colon adenoma in semi-real time. Moreover, because we were able to distinguish normal colonoscopic images from adenomatous colonoscopic images with an accuracy of 99.0% in our pilot study, we are planning to use our dataset in a colon adenoma detection study and we anticipate the construction of a model that can detect and diagnose a colon adenoma simultaneously in real time.

We constructed heatmaps to visualize surface intensity information and understand the classification mechanism of the different CNNs (Fig. 6). This is a promising tool for reviewing the decision made by CNN-CAD. In our study, heatmap locations in A-LGD were consistent across the CNN models, but high intensity lesions in A-HGD were relatively inconsistent, resulting in low classification accuracy compared to the other classes. This may be a result of the diverse features of A-HGD; however, we need to evaluate the heatmaps further to understand and improve the model. CAM not only helps us understand and review the decision of CNN-CAD; it may also help us find unique features of the different classes, thereby providing us with valuable information.

The strength of our model is demonstrated by the fact that, although the images used in the study were not originally captured for the purpose of the study, it achieved a high-level performance. In this retrospective image dataset, some images contained blurring, artifacts, bubbles, stools, or an instrument, which made analysis difficult, and the positions of the lesions in the image frames were not consistent. Thus, in a prospective study or actual usage, the accuracy is expected to be higher than in our study. Furthermore, a well-designed prospective randomized controlled study is required to confirm the feasibility of our model. In addition, because image enhancing technologies, such as NBI, I-scan, or FICE, provide more detailed information about the characteristics of the lesions and deep learning algorithms usually perform better with more data, the application of our model with these technologies is expected to show higher accuracy^38^. Interestingly, the endoscopists tended to miss the correct answer by a narrow margin, that is, their answers were close to the correct ones: they rarely chose CA when the correct classification was A-LGD and vice versa. However, the AI method made large mistakes, albeit rarely, such as classifying CA as normal (Supplementary Tables 2–5). These results support the idea that AI can be used as an assistive technology but cannot replace physicians completely despite its recent advances.

Our study has several limitations. Several clinical characteristics, such as the size of colon adenoma or distance between the lesion and the colonoscope, were not included in our study because of lack of data and the limited number of images used. In addition, the study was conducted in two tertiary hospitals, one for training image set and one for test image set, but a total of two hospitals is not enough number to evaluate the validity of our model, and therefore further studies in collaboration including community-based hospitals are required, and our model needs to be modified for successful application in different hospital settings. Only one image per polyp was used in our study to reduce the overfitting issue, and thus, characteristics that cannot be contained in a single image were neglected. Even though image selection was performed by experts, there are no clear standards for certain criteria so selection bias could not be avoided completely. Because our study was performed retrospectively, its efficacy should and will be supported with a prospective study.

In conclusion, we constructed a CAD system for predicting the histology of colorectal tumors in white light colonoscopic images by applying a deep learning model. Our results showed promising classification results that were similar to those of the experts. Our deep learning model not only can predict the histology of the lesions but also has the potential to identify the lesion simultaneously. Further studies and advancement of our model will help endoscopists accurately detect and diagnose colorectal tumors in real time.

## Supplementary Information

Supplementary Tables.

## Data Availability

The data and analysis tools are available from the corresponding author upon request.

## References

[1] Arnold M (2017). Global patterns and trends in colorectal cancer incidence and mortality. Gut.

[2] Simon K (2016). Colorectal cancer development and advances in screening. Clin. Interv. Aging.

[3] Force UPST (2016). Screening for colorectal cancer: US preventive services task force recommendation statement. JAMA.

[4] Rex DK (2012). Risks and potential cost savings of not sending diminutive polyps for histologic examination. Gastroenterol. Hepatol. (N. Y.).

[5] Byrne MF, Shahidi N, Rex DK (2017). Will computer-aided detection and diagnosis revolutionize colonoscopy?. Gastroenterology.

[6] Ladabaum U (2013). Real-time optical biopsy of colon polyps with narrow band imaging in community practice does not yet meet key thresholds for clinical decisions. Gastroenterology.

[7] Maeda Y (2019). Fully automated diagnostic system with artificial intelligence using endocytoscopy to identify the presence of histologic inflammation associated with ulcerative colitis (with video). Gastrointest. Endosc..

[8] Chen PJ (2018). Accurate classification of diminutive colorectal polyps using computer-aided analysis. Gastroenterology.

[9] Kudo SE (2020). Artificial intelligence-assisted system improves endoscopic identification of colorectal neoplasms. Clin. Gastroenterol. Hepatol..

[10] Krizhevsky A, Sutskever I, Hinton GE (2012). Imagenet classification with deep convolutional neural networks. Adv. Neural Inf. Process. Syst..

[11] Simonyan, K. & Zisserman, A. Very deep convolutional networks for large-scale image recognition. *arXiv preprint* arXiv:1409.1556 (2014).

[12] Szegedy, C., Vanhoucke, V., Iofe, S., Shlens, J. & Wojna, Z. Rethinking the inception architecture for computer vision. in *Proceedings of the IEEE Conference on Computer Vision and Pattern Recognition*, 2818–2826. 10.1109/CVPR.2016.308 (2016).

[13] He, K., Zhang, X., Ren, S. & Sun, J. Deep residual learning for image recognition. in *Proceedings of the IEEE Conference on Computer Vision and Pattern Recognition*, 770–778. arXiv:1512.03385 (2016).

[14] Huang, G., Liu, Z. & Weinberger, K. Q. Densely connected convolutional networks. in *Proceedings of the IEEE Conference on Computer Vision and Pattern Recognition*, 4700–4708. arXiv:1608.06993 (2017).

[15] Deng, J., Dong, W., Socher, R., Li, L. & Li, K. Imagenet: A large-scale hierarchical image database. in *2009 IEEE Conference on Computer Vision and Pattern Recognition*, 248–255. 10.1109/CVPR.2009.5206848 (2009).

[16] Paszke, A. *et al*. PyTorch: An imperative style, high-performance deep learning library. *Adv. Neural Inf. Process. Syst. 32,* 8024–8035. arXiv:912.01703 (2019).

[17] Zhou, B., Khosla, A., Lapedriza, A., Oliva, A. & Torralba, A. Learning deep features for discriminative localization. in *2016 IEEE Conference on Computer Vision and Pattern Recognition *(*CVPR*), 2921–2929. 10.1109/CVPR.2016.319 (2016).

[18] Selvaraju, R. R. et al. Grad-cam: Visual explanations from deep networks via gradient-based localization. in *2017 IEEE International Conference on Computer Vision *(*ICCV*), 618–626. 10.1109/ICCV.2017.74 (2017).

[19] James G, Witten D, Hastie T, Tibshirani R (2013). An Introduction to Statistical Learning with Applications in R, 130.

[20] Le Berre C (2020). Application of artificial intelligence to gastroenterology and hepatology. Gastroenterology.

[21] Ribeiro E, Uhl A, Wimmer G, Hafner M (2016). Exploring deep learning and transfer learning for colonic polyp classification. Comput. Math. Methods Med..

[22] Kominami Y (2016). Computer-aided diagnosis of colorectal polyp histology by using a real-time image recognition system and narrow-band imaging magnifying colonoscopy. Gastrointest. Endosc..

[23] Komeda Y (2017). Computer-aided diagnosis based on convolutional neural network system for colorectal polyp classification: Preliminary experience. Oncology.

[24] Byrne MF (2019). Real-time differentiation of adenomatous and hyperplastic diminutive colorectal polyps during analysis of unaltered videos of standard colonoscopy using a deep learning model. Gut.

[25] Tajbakhsh N, Gurudu SR, Liang J (2015). A comprehensive computer-aided polyp detection system for colonoscopy videos. Inf. Process. Med. Imaging.

[26] Lee CK, Lee SH, Hwangbo Y (2011). Narrow-band imaging versus I-Scan for the real-time histological prediction of diminutive colonic polyps: A prospective comparative study by using the simple unified endoscopic classification. Gastrointest. Endosc..

[27] Yoshida N (2011). Efficacy of magnifying endoscopy with flexible spectral imaging color enhancement in the diagnosis of colorectal tumors. J. Gastroenterol...

[28] Buchner AM (2010). Comparison of probe-based confocal laser endomicroscopy with virtual chromoendoscopy for classification of colon polyps. Gastroenterology.

[29] Mori Y (2013). Comprehensive diagnostic ability of endocytoscopy compared with biopsy for colorectal neoplasms: A prospective randomized noninferiority trial. Endoscopy.

[30] Gross S (2011). Computer-based classification of small colorectal polyps by using narrow-band imaging with optical magnification. Gastrointest. Endosc..

[31] Tischendorf JJ (2010). Computer-aided classification of colorectal polyps based on vascular patterns: A pilot study. Endoscopy.

[32] Ştefănescu D (2016). Computer aided diagnosis for confocal laser endomicroscopy in advanced colorectal adenocarcinoma. PLoS ONE.

[33] Takeda K (2017). Accuracy of diagnosing invasive colorectal cancer using computer-aided endocytoscopy. Endoscopy.

[34] Ignjatovic A (2011). Development and validation of a training module on the use of narrow-band imaging in differentiation of small adenomas from hyperplastic colorectal polyps. Gastrointest. Endosc..

[35] Basford PJ, Longcroft-Wheaton GR, Bhandari P (2013). The learning curve for in-vivo characterisation of small colonic polyps: Number needed to train (NNT) is 200 polyps. Gastrointest. Endosc..

[36] Picot J (2017). Virtual chromoendoscopy for the real-time assessment of colorectal polyps in vivo: A systematic review and economic evaluation. Health Technol. Assess..

[37] Choi Y (2012). Optimal number of endoscopic biopsies in diagnosis of advanced gastric and colorectal cancer. J. Korean Med. Sci..

[38] Utsumi T (2015). Polyp detection, characterization, and management using narrow-band imaging with/without magnification. Clin. Endosc..

[39] Choi, K., Choi, S. J. & Kim, E. S. Computer-aided diagnosis for colorectal cancer using deep learning with visual explanations. in *42nd Annual International Conference of the IEEE Engineering in Medicine and Biology Society*, 1156–1159 (2020) 10.1109/EMBC44109.2020.9176653 33018192

