# Supplementary material for: Prediction of the histology of colorectal neoplasm in white light colonoscopic images using deep learning algorithms
Source: Sci Rep. 2021 Mar 5;11:5311. doi: 10.1038/s41598-021-84299-2 (PMC7935886; doi:10.1038/s41598-021-84299-2)
Supplement: Supplementary file 1 — Supplementary Tables. [file 41598_2021_84299_MOESM1_ESM.pdf]

Prediction of the Histology of Colorectal Neoplasm in White Light Colonoscopic Images Using Deep Learning Algorithm

Seong Ji Choi, Eun Sun Kim, Kihwan Choi

Supplementary Table 1. Classification models pre-trained on ImageNet dataset

	Number of layers	Number of trainable parameters	Top-1 accuracy	Top-5 accuracy	Year
Inception-v3	42	23.9 million	77.4%	93.6%	2015
ResNet-50	50	25.7 million	76.2%	92.8%	2016
DenseNet-161	161	29.0 million	77.6%	93.8%	2017

Supplementary Table 2. Confusion matrix for Inception-v3 for the 10-fold cross-validation. The best validation accuracy model is evaluated. Each entry is the total number of samples predicted to have the row diagnosis label, when the sample was annotated with the column diagnosis label.

		True Labels				
		Normal	LGD	HGD	CA	PPV
Predicted Labels	Normal	994	6	0	0	99.4%
	LGD	4	913	55	10	93.0%
	HGD	1	68	400	63	75.2%
	CA	1	13	45	431	80.0%
	Sensitivity	99.4%	91.3%	80.0%	85.5%	

CA, adenocarcinoma; HGD, high grade dysplasia; LGD, low grade dysplasia; PPV, positive predictive value; s, seconds

Supplementary Table 3. Confusion matrix for ResNet-50 for the 10-fold cross-validation. The best validation accuracy model is evaluated. Each entry is the total number of samples predicted to have the row diagnosis label, when the sample was annotated with the column diagnosis label.

		True Labels				
		Normal	LGD	HGD	CA	PPV
Predicted Labels	Normal	994	7	2	2	98.9%
	LGD	4	908	43	8	94.3%
	HGD	1	75	423	65	75.0%
	CA	1	10	65	429	90.9%
	Sensitivity	99.4%	90.8%	84.6%	85.1%	

CA, adenocarcinoma; HGD, high grade dysplasia; LGD, low grade dysplasia; PPV, positive predictive value; s, seconds

Supplementary Table 4: Confusion matrix for DenseNet-161 for the 10-fold cross-validation. The best validation accuracy model is evaluated. Each entry is the total number of samples predicted to have the row diagnosis label, when the sample was annotated with the column diagnosis label.

		True Labels				
		Normal	LGD	HGD	CA	PPV
Predicted Labels	Normal	997	6	1	0	99.3%
	LGD	2	919	53	12	93.2%
	HGD	0	60	408	57	77.7%
	CA	1	15	38	435	90.0%
	Sensitivity	99.7%	91.9%	81.6%	86.3%	

CA, adenocarcinoma; HGD, high grade dysplasia; LGD, low grade dysplasia; PPV, positive predictive value; s, seconds

Supplementary Table 5: Confusion matrix for CNN-CAD for the 10-fold cross-validation. The best validation accuracy model is evaluated. Each entry is the total number of samples predicted to have the row diagnosis label, when the sample was annotated with the column diagnosis label.

		True Labels				
		Normal	LGD	HGD	CA	PPV
Predicted Labels	Normal	997	5	0	1	99.4%
	LGD	2	922	48	7	94.2%
	HGD	0	65	420	59	77.2%
	CA	1	8	32	437	91.4%
	Sensitivity	99.7%	92.2%	84.0%	86.7%	

CA, adenocarcinoma; HGD, high grade dysplasia; LGD, low grade dysplasia; PPV, positive predictive value; s, seconds

Supplementary Table 6. Diagnostic performance of endoscopists in classification of colon adenoma with KUMC images

	Expert 1	Expert 2	Expert 3	Expert 4	Trainee 1	Trainee 2	Trainee 3	Trainee 4	Trainee 5	Trainee 6
Sensitivity, %	85.00	86.00	84.00	85.00	74.00	77.00	80.00	76.89	81.00	79.00
Specificity, %	95.00	95.33	94.67	95.00	91.33	92.33	93.33	92.14	93.67	93.00
PPV, %	84.99	86.91	84.45	86.62	73.05	76.07	83.24	76.81	81.47	79.70
NPV, %	95.09	95.33	94.73	95.06	91.47	92.57	93.40	92.25	93.94	92.95
Diagnostic time, s	8.89 ± 6.80	8.48 ± 4.91	7.00 ± 3.33	7.46 ± 4.17	11.05 ± 7.41	9.27 ± 6.25	10.61 ± 6.57	8.09 ± 3.78	8.89 ± 6.80	9.40 ± 6.77

NPV, negative predictive value; PPV, positive predictive value; s, seconds

Supplementary Table 7. Diagnostic performance of endoscopists in classification of colon adenoma with HYUMC images

	Expert 1	Expert 2	Expert 3	Expert 4	Trainee 1	Trainee 2	Trainee 3	Trainee 4	Trainee 5	Trainee 6
Sensitivity, %	72.50	73.00	70.00	74.00	59.25	64.75	65.50	64.25	58.25	63.00
Specificity, %	90.92	90.02	90.06	91.33	86.33	88.33	82.07	88.17	86.42	87.50
PPV, %	72.25	70.27	69.07	73.91	59.16	65.64	58.59	66.16	58.77	63.12
NPV, %	90.84	91.09	90.24	91.37	86.41	88.23	85.98	87.97	86.26	87.61
Diagnostic time, s	6.39 ± 3.46	8.92 ± 4.55	8.02 ± 4.13	7.55 ± 4.31	8.05 ± 5.41	8.33 ± 5.11	9.64 ± 5.37	7.11 ± 3.55	8.33 ± 5.13	7.34 ± 4.12

NPV, negative predictive value; PPV, positive predictive value; s, seconds
